# Editorial: Spotlight on aging: anthropological factors impacting physiology, prevention and management of aging conditions

**DOI:** 10.3389/fphys.2023.1331892

**Published:** 2023-11-14

**Authors:** Ahmad Alkhatib

**Affiliations:** ^1^ School of Health and Life Sciences, Teesside University, Middlesbrough, United Kingdom; ^2^ Laboratory of Exercise Biochemistry, College of Kinesiology, University of Taipei, Taipei, Taiwan

**Keywords:** age, longevity, exercise physiology, long term conditions, prevention

Human longevity and the increased life expectancy have come with concerning burdens of ageing related non-communicable diseases (NCDs), including obesity, diabetes, cancer, hypertension, and cardiovascular disease. Although people of all age groups, regions, and countries are affected by NCDs, these conditions are often associated with older age groups (WHO, 2021). Ageing is characterised by physical and mental decline of the human organism, which is a complex interaction between very diverse mechanisms of anthropometry and body composition, metabolic, hormonal, and neuromuscular. For example, most recent discoveries explained ageing development by associating relative bone mass changes and the growing body size with insulin production mechanisms throughout the life course (Lin et al., 2021).

Preventing and managing age-related chronic conditions require deep understanding of anthropological and associated physiological and biomechanical determinants associated with different age clusters and populations across their lifespan. Multiple long term conditions including obesity, diabetes, fatty liver, and cardiovascular disease, are often concurrent in high-risk individuals and should now be jointly addressed through preventative interventions and public health strategies (Alkhatib, 2023). Interventions addressing modifiable lifestyle factors of physical activity and nutrition in high-risk ageing populations continue to provide the most effective risk reduction in various age-related physical, psychological, and biomechanical impairments (Alkhatib, 2023). New research is always needed on innovative physiological intervention approaches, population-based surveys, assessment and implementation methods in the high-risk ageing populations.

This Research Topic of *Frontiers in Physiology* highlighted an important theme of “*Spotlight on Aging: Anthropological Factors Impacting Physiology, Prevention and Management of Aging Conditions*.” Scientific reports on ageing from 16 countries involving 35 researchers (Slovenia, China, Brazil, Poland, Serbia, Croatia, and Germany). Out of 25 manuscripts and abstracts, 11 full submissions were considered, of which six manuscripts were published. Innovative topics included ageing physiology, exercise interventions in women, population surveys, scoping, and systematic reviews.

Population-based surveys provide valuable assessment tools to understand the interaction between environmental and lifestyle determinants associated with age. A large scale survey conducted by Podstawsk et al. assessed 661 Polish women (age >60 years). The survey showed a high prevalence of overweight and gynoid obesity among women (37.2% for body fat percentage and 28.5 kg/m^2^ for body mass index). Higher fat mass and lower fat free mass were negatively correlated with physical activity, health and socioeconomic status. Those findings are in line with previously reported associations of socioeconomic status and indices of all-cause mortality and cardiovascular disease mortality reported in older individuals with type-2 diabetes (Rawshani et al., 2016). Increasing physical activity levels in older women from low socioeconomic and health status is recommended to reduce age-related risks, especially sarcopenia.

Fortunately, the risks of obesity and sarcopenia can be reduced by lifestyle interventions such as increasing physical activity levels in high-risk populations. A 12-week intervention by Špirtović et al. involved 64 healthy older women (34 intervention and 30 control). The program applied a mix aerobic model (55% aerobics and 25% strength and 20% warm up and cool down activities) and achieved significant reduction in anthropometric measures of body mass index (−2.5%) and body fact percentage (−10.59%) waist size (−3.39%), and an increase in muscle mass (2.26%), (all *p* < 0.05). This study showed that a single component active lifestyle intervention is feasible for achieving remarkable risk reduction in age-dependent risks of obesity and sarcopenia in older women.

Ageing research continuously requires updating and developing assessment tools including biomechanical and physiological measurement methods. This exact point was addressed by the work of Pus et al. who systematically reviewed the usefulness of tensiometry, which assess muscle mechanical function, in order to establish reference values for main tensiomyography parameters specific to older adults. Their findings showed that muscle contractile properties can be assessed in older adults with variety of musculoskeletal and vascular conditions including osteoarthritis, arterial stiffness and sarcopenia. Hence the authors concluded that tensiomyography as a non-invasive tool helps assessing neuromuscular function in older adults because of its sensitivity to skeletal muscle composition (especially pre atrophic changes) and responses to muscle quality changes in ageing populations.

Sarcopenia and obesity among older adults are often explained by poor lifestyle physical activity and nutritional habits. An interesting cross sectional study (Teraž et al.) scrutinised sarcopenia and obesity associations with physical activity and nutritional behaviours in 52 older men and women (>65 and <85 years old). Obesity and sarcopenic parameters were measured using Dual Energy X-ray Absorptiometry, muscle strength and physical performance analyses, whereas lifestyle behaviours of sedentariness, physical activity and nutrition were measured using accelerometers and validated questionnaires respectively. Multiple linear regression analysis found no correlation between obesity sarcopenia parameters of body fat or body mass index and the behavioural factors of sedentariness, physical activity, and nutritional habits. The study suggested that higher levels of physical activity should be recommended to prevent obesity and sarcopenic obesity in older adults. Sedentary and physical inactivity were strongly and independently associated with the decline in mental, cognitive, physical, and functional health among older adults in retirement communities (Rosenberg et al., 2016). High adherence to a Mediterranean diet was also reported independently of sarcopenia, and authors explained this dissociation by an inadequate dietary intake, suggesting that preventing sarcopenic obesity should address the excess fat intake rather than simply focus on malnutrition.

Understanding the ageing link with lifestyle physiological, biomechanical and motor control determinants is complex. Systematic reviews provide an excellent tool to decipher such interaction. Hao et al. systematically reviewed the prevention and treatment of Alzheimer’s disease (AD) based on physiological molecular mechanisms of interaction between exercise and motor factors. Physical activity and body composition associations with hippocampal genesis, cognition and aetiology of AD reviewed here makes it a very interesting read. Exercise interventions in humans and animal models showed positive molecular mechanisms adaptations related to AD development. For example, exercise contributes to the stability and restoration of the blood-brain barrier. On a molecular level exercise can activate a number of signalling pathways inhibited by AD (e.g., Wnt and PI3K/Akt signalling pathways) and reactivate the effects of downstream factors regulated by these signalling pathways, thus acting to alleviate autophagic dysfunction, relieve neuroinflammation and mitigate Aβ deposition. Such benefits may be augmented with nutritional polyphenol intake through enhancing the enzymatic functions. Finally, a randomised controlled trial protocol by Sobrinho et al. involved physically inactive elderly women (60–70 years old), who followed 14-week exercise intervention with or without flexibility component. The age-related outcomes included blood pressure, biomechanical and flexibility.

Conclusion: This Research Topic has provided a snapshot on understanding both determinants and the prevention of ageing and associated long term conditions. While studies here showed that lifestyle interventions have a clear advantage in reversing age-related risks, interventions in advanced multiple long-term conditions are needed.
